# Methods in molecular biology and genetics: looking to the future

**DOI:** 10.1186/s13104-023-06298-y

**Published:** 2023-03-02

**Authors:** Diego A. Forero, Vaibhav Chand

**Affiliations:** 1 School of Health and Sport Sciences, Fundación Universitaria del Área Andina, Bogotá, Colombia; 2 Department of Biochemistry and Molecular Genetics, University of Illinois at Chicago, Chicago, USA

**Keywords:** Molecular biology, Molecular genetics, Methods

## Abstract

In recent decades, advances in methods in molecular biology and genetics have revolutionized multiple areas of the life and health sciences. However, there remains a global need for the development of more refined and effective methods across these fields of research. In this current Collection, we aim to showcase articles presenting novel molecular biology and genetics techniques developed by scientists from around the world.

## A brief overview of the development of methods of molecular biology and genetics

Since ancient times, humankind has recognized the influence of heredity, based on familial resemblance, selective breeding of livestock, and climate-adapted crops. Prior to Gregor Johann Mendel’s work in the nineteenth century, there was no clear scientific theory to explain heredity. Mendel’s work remained essentially theoretical until the discovery of DNA and confirmation of its role as the principal agent of heredity in organisms in the twentieth century [1]. In addition, the resolution of the DNA structure paved the way for the invention of the Polymerase Chain Reaction (PCR) (by Kary Mullis), nucleotide synthesis [2] and the Sanger sequencing method [3] which revolutionized the field of genetics and led to the development of several sub-disciplines, including cytogenetics, biotechnology, bioprocess technology, and molecular biology. Automation of Sanger sequencing led to the Human Genome Project in 1990 [1], soon followed by sequencing the complete genomes of numerous other species of flora and fauna [4].

In recent decades, advances in methods in molecular biology and genetics have revolutionized multiple areas of life and health sciences [2]. As a major example from health sciences, PCR-based methods have advanced our understanding of the aetiology of a myriad of acute and chronic diseases, in addition to allowing the diagnosis of multiple disorders [1, 5]. As a recent global application of molecular methods, the PCR-based approaches have led to the processing of hundreds of millions of samples for the analysis of the SARS-CoV-2 virus [6]. In addition, molecular methods have been key for the creation of multiple companies, products and jobs [7].

The development of sequencing technologies and their iterative improvements have been instrumental in advancing the understanding of DNA and RNA, their identification, association with various proteins, their covalent modifications, the function of the genes they carry, and the function of the non-coding portion of DNA and RNA in normal and diseased cells, in pathogenic bacteria and viruses, and in plants [8, 9]. By producing RNA-based vaccines, we were able to combat the recent SARS-CoV2 pandemic. This was made possible by sequencing and *in vitro* nucleotide synthesis technologies [10].

Gene editing technologies, such as restriction endonuclease digestion, transcription activator-like effector nucleases (TALENs), and the clustered regularly interspaced short palindromic repeats (CRISPR-Cas) system, are an additional development in the field of molecular biology that has aided in the understanding of DNA and genes. There is optimism about the use of CRISPR-Cas9 technology in the treatment of a wide variety of diseases, such as cancer, blood-related diseases, hereditary blindness, cystic fibrosis, viral diseases, muscular dystrophy, and Huntington´s disease, due to its precision and its constant improvement, in comparison with other gene-editing technologies [15].

## Need for novel methods in molecular biology and genetics

There is a global need for the development of novel methods for molecular biology and genetics. Particularly, in the area of human health, there is a need for further approaches that facilitate point-of-care molecular analysis (particularly miniaturized and portable platforms), for infectious and non-transmissible diseases [11], the development of more efficient methods for DNA sequencing [3], which facilitate cost-effective genome-wide analysis of patients, among others.

In addition, three key factors would also help push this field forward: additional research comparing the performance of different methods for molecular biology [12], the broader use of reporting standards (such as the Minimum Information for Publication of Quantitative Real-Time PCR Experiments -MIQE-, which describes details of experimental conditions) [13], and the increased participation of scientists from the Global South.

Although older techniques, such as x-ray crystallography, gene cloning, PCR, and sequencing, have been instrumental in the study of various aspects of genetics, these techniques have several limitations that result in gaps, missing links, and incomplete understanding of the genome. Advances in these techniques are needed to fill in these missing pieces of the puzzle to better comprehend genetics and accelerate the discovery of the causes of various genetically linkeddiseases. From a technological standpoint, the accuracy of sequencing and coverage across the genome remain major issues, especially for GC-rich regions and long homopolymer stretches of DNA. Furthermore, the short read lengths generated by the majority of current platforms severely restrict our ability to accurately characterize large repeat regions, numerous indels, and structural variation, rendering large portions of the genome opaque or inaccurate. Fragmentation of the genome for sequencing continues to be a major source of disruption in the continuity of the correct genomic sequence [14, 15].

Recent advances in CRISPR technology provide hope for the medical treatment of cancer and other fatal diseases. Despite significant advances in this field, a number of technical obstacles remain, including off-target activity, insufficient indel or low homology-directed repair (HDR) efficiency, *in vivo* delivery of the Cas system components, and immune responses. This requires a substantial amount of technological advancement or the creation of new, superior methods to combat severe diseases with minimal side effects [14, 16].

## Additional considerations

As high-throughput, automated methods commonly produce very large amounts of data, deeper interaction between wet-lab and dry-lab researchers is required, to facilitate the design of efficient assays [17] and allow effective analysis and interpretation of results. Interdisciplinary collaborations, between biologists, engineers and professionals in the health sciences, might lead to newer and better methods of addressing current and future needs.

Further collaborations between scientists from academia and industry (in addition to researchers from government agencies) [18] would help to facilitate the development of novel methods, and aid in promoting their implementation around the world. For many countries, the main barrier to the broad use of molecular methods is the high cost of equipment and reagents [19]. Strategies aimed at lowering costs would be helpful for multiple institutions around the globe. In terms of intellectual property, fair licensing to institutions in the Global South as well as the implementation of Open Innovation and Open Science policies would be appropriate [20].

## Overview of the current collection

In this current Collection, we are calling for articles showcasing novel methods from molecular biology and genetics, written by scientists from around the world. It is our goal to compile a set of articles that will help to address the challenges faced by the fields of molecular biology and genetics and broaden our understanding of genetic disorders and potential treatment strategies. We invite researchers working on such methods to consider submitting to our collection.

## Data Availability

Not applicable.

## Declarations

### Authors’ information

DAF is a medical doctor, Ph.D. in Biomedical Sciences and Professor and Research Leader at the School of Health and Sport Sciences, Fundación Universitaria del Área Andina (Bogotá, Colombia). He has worked with multiple methods of molecular biology and genetics and is an author of more than 100 articles in international journals, has been peer reviewer for more than 115 international scientific journals, in addition to being part of editorial boards of several international journals. VC is a Research Assistant Professor in the Department of Biochemistry and Molecular Genetics at the University of Illinois at Chicago. His expertise in Biochemistry, Molecular Biology, Genetics, Oncology, and Cancer Biology is extensive. He is an invited reviewer for more than fourteen international peer review journals and is the author of fourteen articles with high impact.
